# Proteolytic Vesicles Derived from *Salmonella enterica* Serovar Typhimurium-Infected Macrophages: Enhancing MMP-9-Mediated Invasion and EV Accumulation

**DOI:** 10.3390/biomedicines12020434

**Published:** 2024-02-15

**Authors:** Alon Nudelman, Anjana Shenoy, Hyla Allouche-Arnon, Michal Fisler, Irit Rosenhek-Goldian, Lior Dayan, Paula Abou Karam, Ziv Porat, Inna Solomonov, Neta Regev-Rudzki, Amnon Bar-Shir, Irit Sagi

**Affiliations:** 1 Department of Immunology and Regenerative Biology, Weizmann Institute of Science, Rehovot 7610001, Israel; alon.nudelman@weizmann.ac.il (A.N.); anjana.shenoy@weizmann.ac.il (A.S.); lior.dayan@weizmann.ac.il (L.D.); inna.solomonov@weizmann.ac.il (I.S.); 2 Department of Molecular Chemistry and Materials Science, Weizmann Institute of Science, Rehovot 7610001, Israel; hyla.arnon@weizmann.ac.il (H.A.-A.); michal.fisler@weizmann.ac.il (M.F.); amnon.barshir@weizmann.ac.il (A.B.-S.); 3 Department of Chemical Research Support, Weizmann Institute of Science, Rehovot 7610001, Israel; irit.goldian@weizmann.ac.il; 4 Department of Biomolecular Sciences, Weizmann Institute of Science, Rehovot 7610001, Israel; paula.aboukaram@weizmann.ac.il (P.A.K.); neta.regev-rudzki@weizmann.ac.il (N.R.-R.); 5 Life Sciences Core Facilities, Weizmann Institute of Science, Rehovot 7610001, Israel; ziv.porat@weizmann.ac.il

**Keywords:** extracellular matrix, *S.* Typhimurium, extracellular vesicles, matrix metalloproteinases, MMP-9, macrophages

## Abstract

Proteolysis of the extracellular matrix (ECM) by matrix metalloproteinases (MMPs) plays a crucial role in the immune response to bacterial infections. Here we report the secretion of MMPs associated with proteolytic extracellular vesicles (EVs) released by macrophages in response to *Salmonella enterica* serovar Typhimurium infection. Specifically, we used global proteomics, in vitro, and in vivo approaches to investigate the composition and function of these proteolytic EVs. Using a model of *S.* Typhimurium infection in murine macrophages, we isolated and characterized a population of small EVs. Bulk proteomics analysis revealed significant changes in protein cargo of naïve and *S.* Typhimurium-infected macrophage-derived EVs, including the upregulation of MMP-9. The increased levels of MMP-9 observed in immune cells exposed to *S.* Typhimurium were found to be regulated by the toll-like receptor 4 (TLR-4)-mediated response to bacterial lipopolysaccharide. Macrophage-derived EV-associated MMP-9 enhanced the macrophage invasion through Matrigel as selective inhibition of MMP-9 reduced macrophage invasion. Systemic administration of fluorescently labeled EVs into immunocompromised mice demonstrated that EV-associated MMP activity facilitated increased accumulation of EVs in spleen and liver tissues. This study suggests that macrophages secrete proteolytic EVs to enhance invasion and ECM remodeling during bacterial infections, shedding light on an essential aspect of the immune response.

## 1. Introduction

The extracellular matrix (ECM) is a grid-like dynamic entity composed of glycoproteins and proteoglycans. It serves as a structural, mechanical, and biochemical platform in addition to mediating intra- and extra-cellular signaling for the surrounding cells [1,2]. Present in all tissues and organs, the ECM is essential for tissue and cell physiology, constantly undergoing remodeling to maintain tissue homeostasis [3]. This process is primarily governed by matrix metalloproteinases (MMPs), a family of Zinc-containing endopeptidases that degrade various ECM components [4,5]. In addition to degradation of structural ECM components, MMPs are also known to cleave various cell surface receptors and other bioactive molecules [6]. Dysregulation of MMPs has been implicated in numerous diseases, including cancer, cardiovascular diseases [7], pulmonary diseases [8], and immunopathologies [9,10,11,12,13,14]. 

MMPs can cause significant damage to host tissues and are therefore tightly regulated [15,16]. Under normal physiological conditions, cells express low levels of MMP transcripts (with the exception of MMP-8 and -9 in neutrophils, which are immediately available for release) and typically require increased gene transcription in order to drive secretion. MMP-9, also known as gelatinase B, is a multidomain enzyme, in which the metal-binding site and the active site form the catalytic domain, inactivated by the aminoterminal propeptide domain [17]. MMP-9 expression is primarily regulated by extracellular signal-regulated kinase (ERK1/2), although other signaling pathways have been identified [12]. For instance, macrophages upregulate MMP-9 expression when exposed to MMP-1, MMP-3, interferon gamma (IFN-γ), and lipopolysaccharide (LPS) [12]. Various signaling molecules have been identified in cells of different origins. Unlike neutrophils, which store MMP-9 in zymogen granules ready for rapid secretion upon inflammatory stimulus [18], macrophages depend on de novo synthesis of MMP-9 prior to secretion, which is a process that takes several hours [19]. 

Once secreted, MMPs are kept in close proximity to the cells [20,21], and their activity is regulated by specific non-covalent inhibitors, the tissue inhibitors of metalloproteinases (TIMPs) [22]. Regulation of MMP-9 is crucial due to its involvement in various physiological processes, but its dysregulation has also been implicated in autoimmune, neoplastic, degenerative, and inflammatory diseases [12]. Increased MMP-9 expression and activity have been reported in a range of lung conditions [23,24,25], various cancers [26,27,28,29,30], immunopathology in infectious diseases [9,10,11], and more [12]. The role of MMP-9 in infectious diseases is particularly interesting and highlights the dual nature of these proteases. MMP-9 is crucial to the normal course of the immune response, facilitating leukocyte migration and invasion to the site of infection or inflammation [31]. It also modulates inflammatory cell recruitment by processing cytokines and chemokines like tumor necrosis factor (TNF) [32], interleukin 1β (IL-1β) [33], interleukin 8 (IL-8) [34], C-X-C motif chemokine ligand 1 (CXCL1) [34], and others. However, dysregulated MMP-9 expression and activity in infectious diseases can cause significant tissue damage [9,10,11,35,36,37,38,39]. Neutrophils and macrophages are major sources of MMP-9 secretion into the tissue, leading to tissue damage both in the physiological immune response and in dysregulated immunopathology [10,11,35,36,37,38,39]. In recent years, researchers have started to report the presence of proteolytic matrix remodeling enzymes, primarily MMPs, within or expressed on the surface of extracellular vesicles (EVs) [40,41]. 

EVs are cargo-bearing lipid bilayer particles naturally released from most cells, considered a common mechanism of molecular exchange and intercellular communication [42]. Cells utilize the various EV biogenesis pathways to control EV cargo selection to communicate specific messages in a selective and regulated manner [43]. EVs can contain different types of cargoes, including cytosolic, membrane and ECM proteins, and nucleic acids [44,45,46,47]. Their cargo is determined by the identity of the parent cell and influenced by physiological and pathophysiological states [48,49]. Certain EV cargo biological molecules, including proteins, serve as EV markers. One such marker is Alix (programmed cell death 6-interacting protein), an accessory protein of the endosomal sorting complexes required for transport (ESCRT) pathway. The ESCRT machinery plays a crucial role in the biogenesis of EVs. Another EV-associated protein is α-tubulin, a cytosolic protein not typically found in the extracellular milieu, indicating the complex nature of EV cargo [43]. 

EVs have been shown to play roles in various pathologies, from cardiovascular diseases [50,51] to neurodegeneration [52,53] and the immune response to bacterial and viral infectious agents [51,54,55]. Most research regarding EVs in the immune response focuses on their role in carrying pro-inflammatory signaling molecules or miRNAs, with little known about their capacity to transport proteolytic enzymes capable of remodeling the extracellular environment or cleaving membrane-bound proteins from adjacent cells.

MMP-9 has been identified in EVs in different physiological and pathophysiological processes, from pro-angiogenic breakdown of capillary membranes and localized proteolysis and degradation of the ECM during metastasis and cellular migration, to Alzheimer’s disease and cancer progression [40,41,56,57]. Despite studies identifying EVs as significant players in the infectious disease setting [51,54,55], little is known about EV-associated ECM remodeling enzymes, and EV-associated MMP-9 in particular, in infectious diseases and the role that they play [41]. Secreted MMP-9 has a dual nature, playing important roles in healthy physiology, but also causing significant tissue damage when its activity is dysregulated. MMP-9 packaged within or bound to the surface of EVs may provide the enzyme with biological and biochemical advantages compared to freely secreted MMP-9. These advantages include potential protection from inactivation by TIMPs, highly localized action, and increased tissue penetration. Furthermore, EV-associated MMP-9 may contribute significantly to the overall levels of the secreted enzyme.

In this study, we utilized a model of *Salmonella enterica* serovar Typhimurium infection in murine macrophages to investigate a population of small EVs. Through comprehensive proteomic analysis, we identified significant alterations in the protein cargo of naïve and *S.* Typhimurium-infected macrophage-derived EVs, particularly a substantial increase in MMP-9 levels. Additionally, we determined that the augmented MMP-9 expression in macrophages exposed to *S.* Typhimurium resulted from the toll-like receptor 4 (TLR-4)-mediated response to bacterial LPS. Tandem co-immunoprecipitation mass spectrometry data strongly suggest the localization of MMP-9 on the EV membrane due to its interaction with EV membrane proteins. We demonstrated that macrophage-derived EV-associated MMP-9 significantly enhanced macrophage invasion through synthetic ECM, and intravenous injection of fluorescently labeled EVs into immunocompromised mice revealed that EV-associated MMP activity substantially increased the accumulation of proteolytic EVs in spleen and liver tissues.

Overall, this study highlights the pivotal role of immune-derived protease-bearing EVs in macrophage invasion and ECM remodeling during bacterial infections, offering valuable insights into the interplay between EVs and the immune response, along with their implications for infectious disease management.

## 2. Materials and Methods 

### 2.1. Cell Lines and Cell Culture

J774A.1 cells (ATCC number TIB-67^TM^, Lot number 70040953, Manassas, VA, USA) were cultured in DMEM medium (Gibco 41965-039, UK), supplemented with penicillin (100 U/mL) and streptomycin (100 mg/mL) (Gibco 15140-122, USA), and 10% fetal bovine serum (FBS) (Sigma F7524-500ML, Mexico). Caco-2 cells were cultured in MEM-Eagle, Earle’s Salts Base (Gibco 11095080, USA), supplemented with penicillin (100 U/mL) and streptomycin (100 mg/mL), and 20% FBS. All cells were maintained in a humidified incubator with 5% CO_2_ at 37 °C and routinely tested and confirmed to be free of mycoplasma contamination.

### 2.2. Salmonella enterica Serovar Typhimurium Infection Assay

J774A.1 were inoculated into 10 cm plates and incubated with heat killed GFP+ *Salmonella enterica* serovar Typhimurium strain SL1344 (HKST) (kindly donated by the Roi Avraham lab, Weizmann Institute of Science) at an MOI of 50, or live GFP+ *S*. Typhimurium (ST) (SL1344) at an MOI of 10 or 50 for 30 min, allowing the bacteria to enter the macrophages, either by infection or phagocytosis. The cells were then washed with phosphate buffered saline (PBS) (Sigma D8537-500ML, UK) supplemented with 30 µg/mL gentamicin (Sigma Aldrich G1272-10ML, Israel) to remove bacteria which had not entered the cells and incubated in EV isolation media supplemented with 15 µg/mL gentamicin. After an incubation of 24 h, EVs were isolated from the EV isolation media by sequential ultra-centrifugation, while the cells were washed with PBS and lysed. Sample protein concentration was determined by bicinchoninic acid assay (BCA) protein assay (Pierce™ BCA Protein Assay Kit). 

### 2.3. EV Purification, Characterization and Analysis

EVs were purified by sequential ultracentrifugation. Cells and debris were removed by centrifugation at 1500× *g* for 20 min. Large microvesicles were then removed by centrifuging the supernatants at 12,000× *g* for 20 min, and the supernatant was then filtered through 0.22 µm filters. Finally, EVs were collected by ultracentrifugation in 39 mL ultracentrifugation tubes at 120,000× *g* for 120 min in a 70 Ti fixed-angle rotor. EVs were resuspended in PBS. Protein concentration was measured by BCA (Pierce, Thermo Fisher Scientific, Waltham, MA, USA). EV size and particle number were analyzed using the NS300 nanoparticle characterization system (NanoSight, Malvern Instruments, Malvern, Worcestershire, UK) and atomic force microscopy (vide infra). 

### 2.4. Western Blot Analysis

EVs were lysed in radioimmunoprecipitation assay (RIPA) buffer (Sigma, 20-188, Burlington, MA, USA), supplemented with a protease inhibitor cocktail (Roche, 04693159001, Germany). An amount of 10 µg of total protein was diluted with sample buffer, run on 10% Tris-glycine gels and transferred onto polyvinylidene fluoride (PVDF) membranes. Membranes were sequentially blocked with 1× PBS containing 5% bovine serum albumin (BSA) (*w*/*v*) and 0.1% Tween 20 (*v*/*v*), incubated with primary antibodies (Recombinant Anti-MMP14 antibody [EP1264Y] (ab51074), Anti-MMP-9 antibody (ab38898), and Anti-ALIX antibody [3A9] (ab117600)) overnight at 4 °C, washed 3 times with 1X PBS containing 0.1% Tween20 (*v*/*v*), incubated with horseradish peroxidase (HRP)-conjugated anti-rabbit (Invitrogen Goat anti-Rabbit IgG (H+L) Secondary Antibody, HRP 31460, Rockford, IL, USA) or anti-mouse (Jackson Immunoresearch, Philadelphia, PA, USA, 115-035-003) secondary antibodies, and washed again to remove unbound antibodies. Bound antibody complexes were detected with ECL. Band intensities and relative densitometry were performed using the ImageJ software v1.54d. 

### 2.5. Gelatin and Collagen Zymographies

8% SDS-PAGE was co-polymerized with 1 mg/mL gelatin or 50 µg/mL collagen type I and then, after loading equal volumes of sample gels, was run under ice-cold conditions at 100 V for one hour. Gels were then incubated with renaturation buffer (2.5% Triton X-100 in distilled H_2_O) for 45 min, then washed twice with developing buffer (50 mM Tris-base, 50 mM Tris-HCL, 0.2 mM NaCl, 5 mM CaCl_2_, distilled H_2_O, and pH adjusted to 7.8–8). Following the wash, gels were submerged in developing buffer and then incubated at 37 °C for 20–24 h. The gels were then stained with Coomassie Brilliant Blue (0.5% *w*/*v*) and imaged using a Bio-Rad GelDoc Imaging System, and clear zones in the gels represented the proteolytic activity.

### 2.6. Atomic Force Microscopy

A freshly cleaved mica surface was incubated with 10 mM MgCl_2_ solution for 2 min, then rinsed twice with 100 μL PBS, 50 µL of EV solution (10^11^ EVs) from either naïve macrophages, or macrophages infected with *S*. Typhimurium at an MOI of 50. The EVs were isolated as described above and further purified by precipitation out of a sucrose cushion (4 h at 100,000× *g* on 20% sucrose). The purified EV solution was deposited on the Mg-modified mica for 15 min of adsorption. Prior to scanning, the sample was washed once with 100 µL PBS, then 100 µL fresh PBS was added. Washing was performed carefully to avoid drying the sample. AFM imaging was performed on a JPK Nanowizard III AFM microscope (Bruker Nano GmbH, Berlin, Germany) in QI mode. Measurements were con-ducted with a qp-BioAC-Cl probe, (Nanosensors, Neuchâtel, Switzerland), spring constant ≈ 0.06 N/m. Image analysis was performed using Gwyddion [58] and JPK-SPM data processing software version 6.2.172.

### 2.7. Co-IP and Mass Spectrometry

Co-immunoprecipitation (Co-IP) was performed according to the Invitrogen DynabeadsTM Protein G user guide. A total of 50 µL (1.5 mg) DynabeadsTM was washed with PBS with 0.02% Tween 20, then incubated with 10 µg of either anti-MMP-9 antibody (abcam–ab38898) or InVivoMAb mouse IgG1 isotype control, unknown specificity (Catalog #BE0083), for 10 min at room temp. The bead-antibody complex was washed with PBS with 0.02% Tween 20, the supernatant removed, and 1.3 mg of EVs from J774A.1 macrophages infected with *Salmonella* Typhimurium at an MOI of 50 bacteria per cell, lysed with 10X RIPA, was added. The bead-antibody complex was incubated with the EV lysate overnight at 4 °C. Immunoprecipitated complexes on the beads were washed three times with buffer I (0.05% IGEPAL-CA-630 (NP-40), 150 mM NaCl, 50 mM Tris–HCl (pH 7.5)) and three times with buffer II (150 mM NaCl, 50 mM Tris–HCl (pH 7.5)). The complexes were eluted from the beads at room temperature for 2 h with 100 μL elution buffer I (2 M urea, 50 mM Tris–HCl -pH 7.5, 1 mM DTT, and 0.4 μg sequencing grade trypsin) and 100 μL elution buffer II (2 M urea, 50 mM Tris–HCl pH 7.5, and 5 mM iodoacetamide) and digested overnight at room temperature. The peptides were acidified with 0.1% trifluoroacetic acid and desalted with C18 stage tips [59]. Peptides were eluted using 80% acetonitrile, vacuum-concentrated and loaded onto a Waters ACQ M-Class HSS T3 column (1.8 µm, 75 µm × 250 mm) coupled to a Waters nanoACQUITY ultra-performance liquid chromatography (UPLC) and Q Exactive HF mass spectrometer. Peptides were separated using a 180-min linear gradient of water and acetonitrile. Raw files were analyzed with MaxQuant [60] software (version 1.5.3.36) with the built-in Andromeda search engine [61]. Bioinformatics analyses were performed using the Perseus program [62]. To identify the proteins that interact specifically with MMP-9, log2 protein intensities of IgG were compared to MMP-9 samples and proteins showing MMP-9/IgG log_2_ ratio > 4 were selected as the interactors. A protein–protein interaction network was created in Cytoscape (version 3.10.0) [63] and Gene Ontology (GO) annotations for proteins were imported from StringDB [64].

### 2.8. Transwell Invasion Assay

GFR Matrigel (Corning product number 354230, Bedford, MA, USA) was thawed on ice, then diluted 1:3 in cold serum-free DMEM. A total of 6.5 mm Transwell inserts with 8.0 µm pores (Corning product number 3422) was coated in 75 µL of the diluted Matrigel and incubated for 30 min at 37 °C. EVs (10 µg) derived from macrophages infected with *Salmonella* Typhimurium at an MOI of 50 bacteria per cell were pre-incubated for 2 h with either PBS, 0.1 µM SDS-3 (prepared in-house), or 1 µM Marimastat (Sigma-Aldrich M2699). The Matrigel-coated Transwells were then treated for 4 h with 100 µL of EVs from the pre-incubated samples, or with 100 µL of EVs (10 µg) derived from naïve macrophages pre-incubated in PBS. The media containing the EVs were removed, and 10^5^ naïve macrophages were inoculated onto the top half of the Matrigel-coated Transwell insert. In the bottom half of the Transwell insert, the media were supplemented with 50 nM of recombinant complement factor C5a (Peprotech Catalog Number: 300-70) and incubated for 36 h. The Matrigel-coated Transwell inserts were removed, washed, and the Matrigel physically extracted from the top half of the Transwell insert. The Transwell insert was then fixed in 4% PFA and stained with crystal violet solution. The invading macrophages were imaged using a Widefield microscope with a DMI8 platform. The number of stained macrophages that had invaded into the bottom half of the Transwell insert were quantified using ImageJ software v1.54d.

### 2.9. EV Labeling with DiR and Retro-Orbital Injection

EV particle concentrations were counted using nanoparticle tracking analysis (NTA) and normalized to approximately 5 × 10^11^ EVs / mL in 0.22 µM-filtered PBS. DiR (DiIC18(7) (1,1′-Dioctadecyl-3,3,3′,3′-Tetramethylindotricarbocyanine Iodide), Invitrogen, catalog number D12731) was added to a final concentration of 15 µM in 400–500 µL, vortexed, incubated for 1 h at room temperature (protected from light), and vortexed every 20 min. After incubation, the samples were transferred to Vivaspin 50 kDa cutoff spin columns and centrifuged at 14,000× *g* for 5 min. If 5 min was insufficient to reduce the sample volume to approximately 100 µL, the previous step was repeated. The flow-through was discarded and 500 µL PBS was added to wash the spin column. This step was repeated a total of 3 times. After the 3rd spin, ~120 µL PBS was collected from the spin column into a fresh tube. The samples were then taken again to NTA to check particle concentrations. After normalizing, 4 × 10^10^ EVs from each sample were injected retro-orbitally into male immunodeficient Hsd:Athymic Nude-Foxn1nu mice. After 24 h, the mice were perfused with 4% paraformaldehyde (PFA) in PBS. The brain, lungs, heart, liver, spleen, kidneys, and intestine were removed from each mouse and kept in 4% PFA overnight at 4 °C. The following day, the organs were imaged in IVIS using Aura Spectral Imaging v4.0.8, at wavelengths corresponding to DiR (excitation 754 nm, emission 780 nm) using exposures of 6–30 s at medium binning. Fluorescence intensity was analyzed using the FIJI ImageJ program v1.54d.

### 2.10. Mass Spectrometry

EV samples were lysed with 100 µL of 8 M urea. Following protein determination, 10 µg of the protein was reduced with TCEP to a final concentration of 10 mM at room temperature for 30 min. Samples were then alkylated with CAA to a final concentration of 40 mM for 30 min at room temperature. Samples were diluted with 50 mM ammonium bicarbonate buffer to obtain a final urea concentration of <1 M. Samples were digested overnight with Lys-C-Trypsin mix (1:100 enzyme: protein ratio) and trypsin (Promega V5073, USA; 1:50 enzyme: protein ratio). Peptides were desalted on C18 stage tips, vacuum dried and resuspended in 2% acetonitrile, 0.1% TFA. The peptide samples were analyzed in the De Botton Protein Profiling Institute of the Nancy and Stephen Grand Israel National Center for Personalized Medicine, Weizmann Institute of Science. Peptides were loaded onto a Waters ACQ M-Class HSS T3 column (1.8 µm, 75 µm × 250 mm) coupled to a Waters nanoACQUITY UPLC and Q Exactive HF mass spectrometer. Peptides were separated using a 180-min linear gradient of water and acetonitrile.

### 2.11. Mass Spectrometry Data Analysis

Raw files were analyzed with MaxQuant software (version 1.5.3.36) with the built-in Andromeda search engine. MS/MS spectra were searched against Mus Musculus FASTA files from the Uniprot database, a reverse decoy database, and common contaminants. The peptide search included cysteine carbamidomethylation as a fixed modification, and N-terminal acetylation and methionine oxidation as variable modifications. Trypsin was selected as the specified protease and a maximum of two missed cleavages were allowed. A false discovery rate cutoff of 1% was applied at both the protein and PSM identification levels. For relative quantification we enabled iBAQ normalization.

Bioinformatics analyses were performed using the Perseus program v1.6.2.1. The protein groups were filtered to remove potential contaminants, peptides matched to the reverse decoy database, and proteins only identified by a modification site resulting in 3225 quantified proteins. Log2 data were filtered to retain only proteins with 70% valid values across samples. Missing data were overcome by imputing values based on normal distribution with a width of 0.3 and a downshift of 1.8 standard deviations. Differentially expressed proteins between naïve and infected macrophage EVs were extracted by performing a paired Student’s *t*-test (permutation-based FDR 0.05). Gene annotations, including GOBP, GOMF, GOCC, and KEGG pathway, were added from Uniprot and a 1D annotation enrichment test was performed on the total protein intensity across all samples (Benjamini Hochberg FDR, q-value < 0.02). Matrisome proteins were annotated based on the matrisome definition [65,66].

### 2.12. Animal Care

Male immunodeficient Hsd:Athymic Nude-Foxn1nu mice (Envigo) were used in the experiments. All animal studies were approved in accordance with the Weizmann Institute’s Animal Care and Use Committee (IACUC) guidelines and regulations (approval number 00580120-3). All animals were kept in a daily controlled room at the Weizmann Institute of Sciences animal facility with a surrounding relative humidity level of 50 ± 10% and a temperature of 22 ± 1 °C, with a 12/12 cycle of dark and light phases.

### 2.13. Statistics

All numerical data are presented as mean ± standard deviation (s.d.). Statistical analysis was performed using GraphPad Prism software v10.2. 0 (GraphPad Software Inc., Boston, MA, USA) or Excel (Microsoft^®^ Excel^®^ 2021 MSO (Version 2401 Build 16.0.17231.20194) 64-bit). Comparison of two groups was analyzed by a two-tailed Student’s *t* test. A *p*-value of 0.05 and below was considered significant: * *p*-value < 0.05, ** *p*-value < 0.01, *** *p*-value < 0.001, and **** *p*-value < 0.0001.

## 3. Results

### 3.1. Establishment and Characterization of an In Vitro Model of Macrophage-Derived EVs

**Figure 1:**
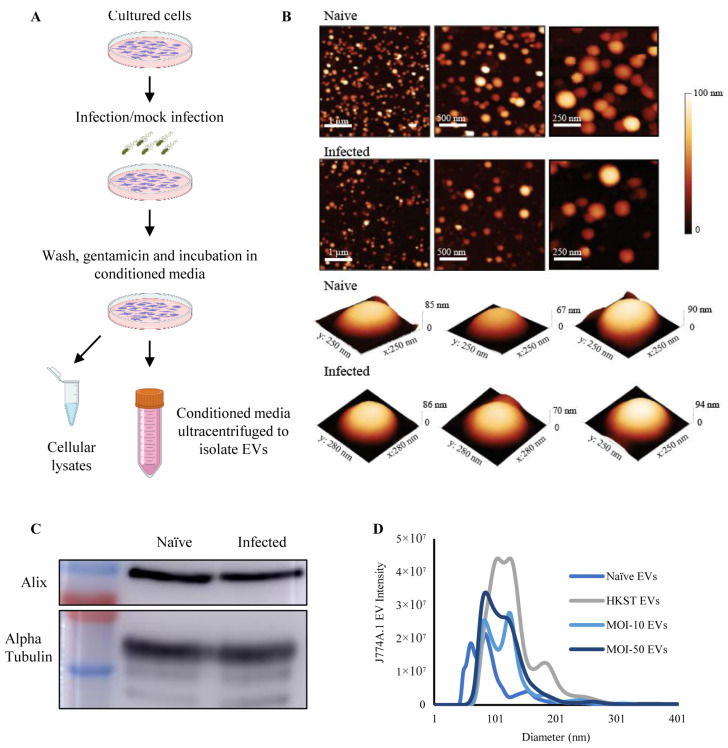
Establishment and characterization of an in vitro model of macrophage-derived EVs. (**A**) Workflow for extracellular vesicle isolation. (**B**) Representative AFM images of macrophage-derived EVs from naïve and *S*. Typhimurium-infected macrophages at different magnifications, along with representative 3D AFM images of single EVs from each sample. (**C**) Representative Western blot of EV-associated Alix and α-tubulin from naïve and *S*. Typhimurium-infected (MOI-50) macrophages. (**D**) Size distribution of macrophage-derived EVs from naïve cells, or cells exposed to heat-killed *S.* Typhimurium (HKST), or live *S.* Typhimurium at an MOI of 10 or 50. Each condition was performed in triplicate and measured using NTA.

To investigate macrophage-derived EVs in host-pathogen interactions, we first established an in vitro model (Figure 1A) utilizing a differentiated and adherent murine macrophage cell line (J774A.1), further detailed in the Methods section. Three million cells were seeded on 10 cm plates and subjected to either heat-killed *S.* Typhimurium or live *S.* Typhimurium infection at a multiplicity of infection (MOI) of 10 or 50 bacteria per cell. Following incubation, EVs were isolated from the conditioned media through sequential centrifugation and ultracentrifugation. EVs were visualized using atomic force microscopy (AFM) after additional purification on a 20% sucrose cushion. All other measurements were conducted on EVs isolated through sequential ultracentrifugation without sucrose cushioning, to prevent a reduction in EV yield. AFM measurements (Figure 1B) demonstrated that the EVs from both the naïve and MOI-50-infected macrophages exhibited a spheroidal shape, with average diameters of 109.9 ± 39.9 and 121 ± 36.4 nm, respectively. To further validate the EVs, they were analyzed by Western blotting for the EV marker proteins Alix, an ESCRT pathway protein, and the cytosolic protein α-tubulin (Figure 1C). Western blotting confirmed the presence of Alix and α-tubulin in EVs from both naïve and MOI-50-infected cells.

Macrophage-derived EVs were analyzed using nanoparticle tracking analysis (NTA). NTA of the macrophage-derived EVs revealed a significant shift in size distribution upon exposure or infection with heat-killed or live *S.* Typhimurium. Mock-infected or infected macrophages secreted EVs with increased diameters (Figure 1D and Figure S1A). Naïve macrophage-derived EVs exhibited an average diameter of 100.3 ± 10.6 nm, whereas EVs from *S.* Typhimurium-infected macrophages had average diameters of 135.4 ± 1.9, 123.6 ± 0.7, and 121.5 ± 2.8 nm (HKST, MOI-10, and MOI-50, respectively) (Supplementary Figure S1A). The number of EVs secreted per cell was calculated using NTA measurements (Supplementary Figure S1B), revealing that upon exposure to either live or heat-killed *S.* Typhimurium, macrophages secreted 2–4-fold more EVs.

### 3.2. Bulk Proteomics of EVs Derived from Naïve or S. Typhimurium-Infected Macrophages Show Enrichment of EV Marker Proteins and Upregulation of MMP-9

**Figure 2:**
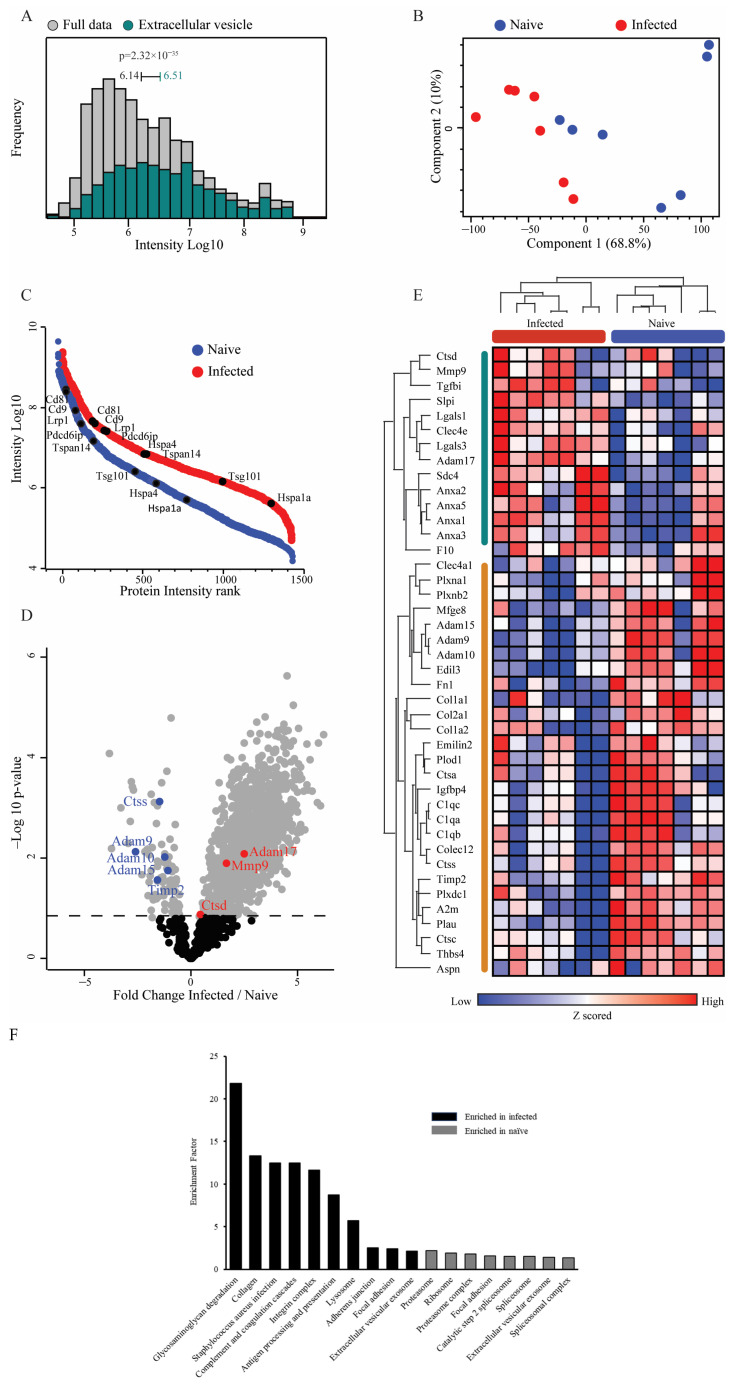
Bulk proteomics of EVs derived from naïve or *S.* Typhimurium-infected macrophages reveals enrichment of EV marker proteins and upregulation of MMP-9. (**A**) Histogram of protein expression intensities in the dataset (grey) compared to the exosome-specific proteins from GOCC (color) demonstrates a significant enrichment of exosome proteins in this dataset (1D annotation enrichment, FDR = 0.02). Median expression intensities and *p*-values are indicated. (**B**) Principal component analysis of naïve and infected macrophage EV protein content shows good separation between groups on the first component. (**C**) EV marker proteins in naïve and infected macrophage EV proteomes. Rank plots showing protein abundances of selected marker proteins. Abundances are depicted as median logarithmic iBAQ protein intensities. (**D**) Volcano plot highlights significantly changing proteins (grey, permutation-based FDR 5%) between naïve and infected EV proteomes. Proteases from the matrisome list are highlighted. (**E**) Heatmap of 42 differentially expressed matrisome proteins. Proteins were selected using a paired Student’s *t*-test with FDR 5% between matched samples and then filtered to retain only the matrisome proteins. (**F**) Pathway enrichment analysis of EV-associated proteins.

EVs from naïve (nEVs) or *S*. Typhimurium-infected (iEVs) macrophages were lysed, and their protein content was analyzed using mass spectrometry. Analysis of the host-derived total protein dataset of both populations showed significant enrichment of EV-associated proteins, regardless of whether the EVs were derived from naïve or *S*. Typhimurium-infected macrophages. This demonstrated the capture of a significant number of EV marker and EV-associated proteins (Figure 2A). Principle component analysis demonstrated clear separation on the first component, indicating distinct compositions of host-derived protein cargo between EVs from naïve macrophages and those from *S*. Typhimurium-infected macrophages (Figure 2B). Presented by their relative abundance, 2555 proteins were commonly identified in the nEV samples and 3124 in the iEVs, including numerous EV marker proteins, e.g., CD9, CD81, PDCD6IP (Alix), and tetraspanin 14 (TSPAN14) (Figure 2C). Furthermore, 1192 proteins displayed differential expression between the naïve and *S.* Typhimurium-infected macrophage-derived EVs, demonstrating significant differences in protein cargo between the two groups (Figure 2D).

Remarkably, examination of matrisome proteins [65,66] demonstrated significant increases in the abundance of the proteases’ matrix metalloprotease (MMP)-9 and in the disintegrin and metalloprotease (ADAM)-17 in the iEVs compared to the nEVs (Figure 2D,E). Conversely, proteases such as ADAM-9, ADAM-10, ADAM-15, and cathepsin S (CTSS) were downregulated, underscoring the selective enrichment of MMP-9 and ADAM-17 in iEVs (Figure 2E). Additionally, TIMP-2, one of the endogenous inhibitors of MMP-9, exhibited significant downregulation, supporting dysregulation of MMP-9 activity. Pathway enrichment analysis highlighted numerous pathways that were upregulated in the infected samples (Figure 2F). Notably, the two most highly enriched pathways in the infected EVs were glycosaminoglycan-degradation- and collagen-associated pathways, both closely linked to the ECM and ECM remodeling. This pathway analysis further indicates the substantial involvement of ECM remodeling enzymes within the iEVs in their functions. To confirm that MMP-9 was associated with EVs, EVs from both naïve and *S*. Typhimurium-infected macrophages were centrifuged for 4 h at 100,000× *g* on top of a cushion of 20% sucrose. Western blotting of 10 µg of protein from the precipitate of the sucrose cushion from both naïve and *S.* Typhimurium-infected macrophages demonstrated the presence of MMP-9 and Alix, with a noticeable increase in MMP-9 band intensity in the *S*. Typhimurium-infected samples (Supplementary Figure S2).

Notably, numerous immune-related proteins were enriched in the *S*. Typhimurium-infected EVs, including TLR-2, intercellular adhesion molecule 1 (ICAM-1), CD14, and complement C3 (C3). These immune-related proteins serve various immuno-stimulatory functions, from ICAM-1 enhancing leucocyte transmigration into tissues, to TLR-2 and CD14 acting as receptors for pathogen-associated molecular patterns (PAMPs), potentially increasing the activation of cells that uptake the EVs, and C3, which is directly involved in complement system activation. The presence of these proteins in iEVs underscores the potential involvement of pro-inflammatory processes in various infectious diseases, consistent with findings in other published studies.

### 3.3. Macrophage-Derived EVs Exposed to Live or Dead S. Typhimurium Demonstrate Increased MMP-9 Activity

**Figure 3:**
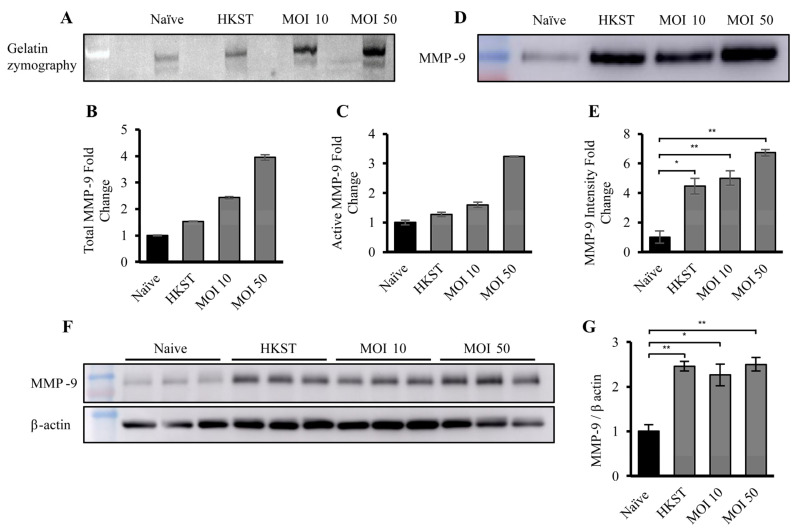
Macrophage-derived EVs exposed to live or dead *S*. Typhimurium demonstrate increased MMP-9 expression and activity. (**A**) Representative gelatin zymography of EVs derived from naïve, HKST, MOI-10-, and MOI-50-infected macrophages, (**B**) densitometry analysis representing both active and pro- MMP-9 (*n* = 2), and (**C**) densitometry analysis representing only active MMP-9, shown as fold change relative to the naïve condition (*n* = 2). The amount of degraded gelatin was quantified using ImageJ v1.54d. (**D**) Representative Western blot of EV-associated MMP-9 from the different conditions and (**E**) densitometry analysis of MMP-9 from EVs isolated from naïve, HKST, MOI-10-, and MOI-50-infected macrophages. Each condition was performed in triplicate. (**F**) Representative Western blots of MMP-9 and β-actin from macrophage cell lysates and (**G**) densitometry analysis of relative MMP-9 amounts from naïve, HKST, MOI-10-, and MOI-50-infected macrophages. The EV samples were normalized to 10 µg total protein per well by BCA analysis protein. Each condition was performed in triplicate. * *p* < 0.05, ** *p* < 0.01.

To assess the proteolytic potential of the macrophage-derived EVs, an in-gel zymography was performed (Figure 3A). Each lane of a gelatin gel was loaded with 10 µg of EVs, and in-gel zymography was employed to compare proteolytic activities. The results revealed an increase in gelatin degradation at ~90 and ~100 kDa, corresponding to the active (92 kDa) and latent (102 kDa) forms of MMP-9, respectively. The densitometry of the 90 kDa band (Figure 3C) reflected a significant increase in active MMP-9 levels, while the analysis of two bands (Figure 3B) demonstrated a noticeable increase in total MMP-9 in EVs from macrophages exposed to heat-killed or live bacteria. To confirm the presence of MMP-9, EVs from naïve, HKST, MOI-10-, and MOI-50-infected macrophages, and the corresponding cellular lysates, were Western blotted and densitometric quantification was carried out using ImageJ v1.54d (Figure 3D–G). The Western bolt analysis revealed that MMP-9 expression was increased ~4-fold in the HKST and MOI-10 EVs and ~6-fold in the MOI-50 EVs (Figure 3D,E), confirming the results of in-gel zymography. The cellular lysates were also subjected to Western blotting for MMP-9 and β-actin (loading control), followed by quantification (Figure 3F,G). The results showed a 2.5-fold increase in total MMP-9 levels in macrophages exposed to heat-killed *S.* Typhimurium or infected with live *S.* Typhimurium at an MOI of 10 or 50.

Notably, the increase in EV-associated proteolytic activity, and the elevation of MMP-9 levels in both EVs and cells occurred in the samples exposed to both heat-killed *S.* Typhimurium and live bacteria. This observation suggests that the bacteria itself did not cause the upregulation of MMP-9 expression or proteolytic activity by manipulating cellular biology; instead, it was a bacterial component responsible for this phenomenon.

### 3.4. TLR-4 Pathway Regulates Upregulation of MMP-9 Expression and Activity in Macrophages and Macrophage-Derived EVs in Response to Gram-Negative Bacteria

Since *S.* Typhimurium is a Gram-negative bacteria coated in LPS, a potential regulator of the increase in MMP-9 expression and activity in macrophages and macrophage-derived EVs is the TLR-4, a pathogen-recognition receptor (PRR) for LPS. TLR-4 is primarily expressed by innate immune cells, such as M1 macrophages, monocytes, and neutrophils, and is responsible for recognizing LPS, triggering either the MyD88-dependent or MyD88-independent pathway, leading to nuclear factor kappa B (NFκB) or interferon regulatory factor 3 (IRF3) activation, respectively. Moreover, TLR-4 has previously been documented to regulate MMP-9 expression in other cell types [67,68,69]. To investigate the pathway regulating MMP-9 activation, we utilized two inhibitors: resatorvid (TAK-242), which inhibits intracellular signaling [70] of TLR-4, and ammonium pyrrolidine dithiocarbamate (APDC), an inhibitor of IκB phosphorylation, blocking NFκB translocation into the nucleus [71].

**Figure 4:**
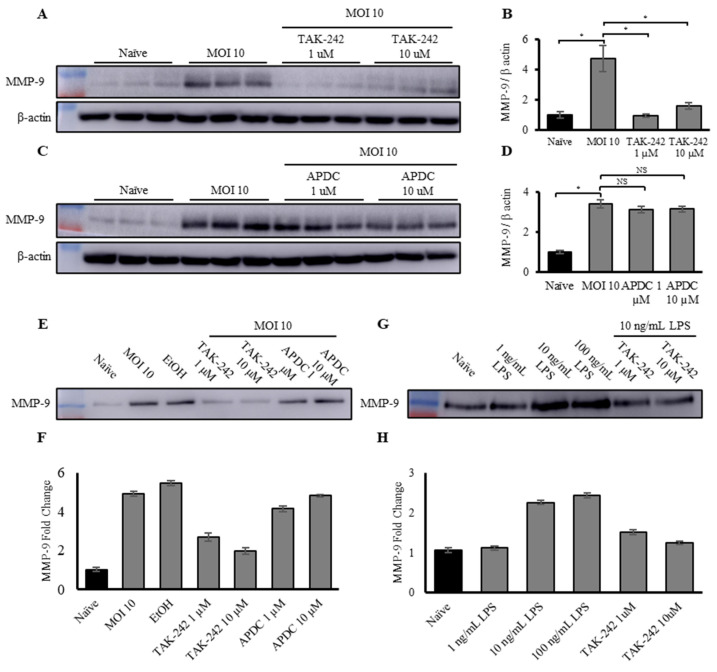
Upregulation of MMP-9 expression and activity in macrophages and macrophage-derived EVs in response to exposure to Gram-negative bacteria is controlled by the TLR-4 pathway. (**A**) Representative Western blot of total MMP-9 and β-actin and (**B**) densitometry analysis of total MMP-9 in naïve, MOI-10-infected macrophages, and macrophages pre-treated with 1 and 10 µM TAK-242 then infected with *S.* Typhimurium at an MOI of 10, normalized to β-actin. Each condition was performed in triplicate. (**C**) Representative Western blot of total MMP-9 and β-actin and (**D**) densitometry analysis of total MMP-9 in naïve, MOI-10-infected macrophages, and macrophages pre-treated with 1 and 10 µM APDC then infected with *S.* Typhimurium at an MOI of 10, normalized to β-actin. Each condition was performed in triplicate. (**E**) Representative Western blot and (**F**) densitometry analysis of total MMP-9 in EVs derived from naïve, MOI-10-infected macrophages, and macrophages pre-treated with 1 and 10 µM TAK-242, or 1 and 10 µM APDC, then infected with *S.* Typhimurium at an MOI of 10, normalized by BCA (*n* = 2). (**G**) Representative Western blot and (**H**) densitometry analysis of total MMP-9 in EVs derived from naïve macrophages and macrophages exposed to either 1, 10, or 100 ng/mL *E. coli* LPS, or pre-treated with 1 or 10 µM TAK-242, then exposed to 100 ng/mL LPS, normalized by BCA (*n* = 2). * *p* < 0.05, NS specify no significant difference between treatments.

J774A.1 cells were pre-treated with 1 or 10 µM TAK-242 or APDC for 1 h prior to infection with live *S.* Typhimurium at an MOI of 10. Western blotting was conducted on cellular lysates to measure MMP-9 and β-actin levels (Figure 4A,C). MMP-9 levels were normalized to β-actin and quantified by densitometry analysis for total MMP-9 (Figure 4B,D). The results showed that pre-treatment with TAK-242 almost completely abrogated the MMP-9 expression caused by infection with *S.* Typhimurium. However, pre-treatment with APDC did not reduce cellular expression of MMP-9 in the infected sample compared to the uninfected control, as shown by Western blotting (Figure 4E,F). Pre-treatment with TAK-242, the TLR-4 signaling inhibitor, also significantly reduced the expression of MMP-9 in macrophage-derived EVs. However, similar to the effect observed in the cells, pre-treatment with APDC, the IκB phosphorylation inhibitor, did not show any reduction in macrophage-derived EV-associated MMP-9 (Figure 4G,H). These results clearly show that TLR-4, the innate immune receptor for bacterial LPS, regulates the increase in both macrophage cellular and macrophage-derived EV-associated MMP-9 expression. This strongly suggests that increased expression of MMP-9 by macrophages is a direct response to bacterial infection. Given that APDC did not reduce MMP-9 expression levels in either cellular or EV-associated MMP-9, it is possible that MMP-9 upregulation is controlled through the Myd-88-independent signaling pathway, culminating in activation of IRF-3, rather than NFκB. However, this requires further study, and IκB phosphorylation needs to be examined. These results are consistent with other reports, showing that one of the mechanisms regulating MMP-9 expression and activation is LPS and TLR-4 signaling [68,69,72].

To further investigate the TLR-4–LPS crosstalk, and to extend the findings beyond *S*. Typhimurium to a general Gram-negative response, macrophages were treated with a dose response of LPS (1, 10, and 100 ng/mL) from *E. coli*, another Gram-negative bacteria, or pre-treated with TAK-242 (1 and 10 µM for 1 h prior to exposure to LPS) and then exposed to the LPS. Western blotting and subsequent analysis (Figure 4G,H) showed that exposure of the macrophages to LPS alone led to a dose-dependent increase in EV-associated MMP-9 expression. Similar to the *S.* Typhimurium findings, pre-treatment with TAK-242 negated the increase in EV-associated MMP-9 in response to exposure to *E. coli* LPS.

### 3.5. MMP-9 Co-Localizes to the EV Membrane via Binding to EV-Associated Membrane Proteins

**Figure 5:**
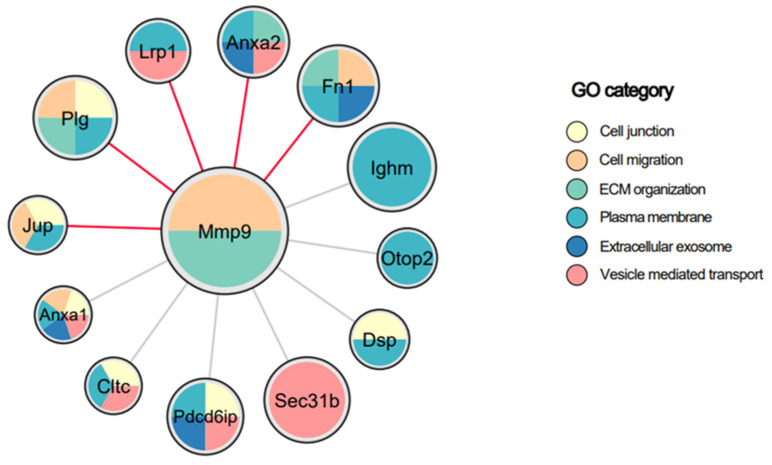
Protein–protein interactions of EV-associated MMP-9, filtered for membrane association. EV lysates were used for immunoprecipitation using antibodies against MMP-9 or IgG. The immune complexes were subjected to mass spectrometry analysis. Membrane proteins showing log2ratio > 4 in MMP-9 compared to IgG are presented. Each node is a protein precipitated with MMP-9 and node size is based on the fold change of binding to MMP-9 over IgG. Node colors are based on the gene ontology (GO) category as indicated. Red edge color indicates known interactions from StringDB.

To probe the localization of MMP-9 within or on the membrane of the EVs, Co-IP followed by mass spectrometry was performed. Magnetic protein G beads were attached to anti-MMP-9 antibody, or anti-IgG for control, and incubated with a lysate of EVs from macrophages infected with *S.* Typhimurium at an MOI of 50. After incubation, the bead-antibody-sample complex was washed and then analyzed by mass spectrometry and bioinformatics analyses. To identify the proteins specifically interacting with MMP-9, log2 protein intensities of MMP-9 were compared to those from IgG samples and a stringent cut-off was applied. Only proteins that exhibited more than a four-fold enrichment in the MMP-9 over IgG pulldown were selected as interactors (Supplementary Table S1). Pathway annotations for MMP-9 and interacting partners filtered for membrane association were imported from StringDB and the network was visualized in Cytoscape (version 3.9.1) (Figure 5). This data revealed two known cell-surface-associated binders of MMP-9, specifically the low-density lipoprotein receptor related protein 1 (LRP-1), a transmembrane-domain receptor, and fibronectin [73,74,75]. These data strongly indicate that macrophage-derived EV-associated MMP-9 is bound to the EV membrane. Notably, annexin A2 and fibronectin have been reported to be expressed on the surface of EVs, while LRP-1 was detected on the surface of EVs for the first time [76,77]. Other proteins identified by Co-IP, while not previously reported to bind MMP-9, suggest that MMP-9 is associated with the plasma membrane by binding to numerous cell-membrane proteins, forming multiprotein complexes, with many of these individual proteins yet to be identified [73,74,78,79,80,81].

### 3.6. S. Typhimurium-Infected Macrophage-Derived EVs Do Not Affect Bacterial Growth or Infectivity

To assess the potential antibacterial activity of EVs, an EV dose response assay was performed. Live *S.* Typhimurium was incubated with a dose response (25 µg/mL to 0.78 µg/mL) of EVs from naïve macrophages (nEVs) or from *S.* Typhimurium-infected macrophages (iEVs) for 22 h at 37 °C, and optical density (600 nm) was read every 20 min (Supplementary Figure S3A). A positive dose response was not observed in either the nEV or the iEV dose response assay (Supplementary Figure S3A). Incubation with both nEVs and iEVs at the low dose of 0.78 µg/mL resulted in a decrease in both maximum bacterial growth and maximum rate of bacterial growth. However, at the highest dose of 25 µg/mL this effect was not observed (Supplementary Figure S3B,C). Furthermore, at both the highest and low doses of EVs, there was no significant difference in maximum growth or rate of growth between the bacteria incubated with the nEVs or iEVs (Supplementary Figure S3B,C).

Additionally, in order to test if EVs secreted by *S.* Typhimurium-infected macrophages affected the bacteria’s infectivity, non-phagocytic Caco-2 epithelial cells were pre-incubated with nEVs or iEVs. After the incubation, the cells were infected with an MOI of 50 bacteria/cell of GFP^+^ *S.* Typhimurium. The percentage of infected (GFP^+^) cells was quantified using ImageStream analysis. The results demonstrated that neither treatment with EVs from nEVs nor iEVs had any significant effect on the number of epithelial cells infected with *S*. Typhimurium (Supplementary Figure S3D).

### 3.7. EV-Associated MMP-9 Derived from S. Typhimurium-Infected Macrophages Increases Macrophage Invasion through Matrigel, a Simulated Basement Matrix

**Figure 6:**
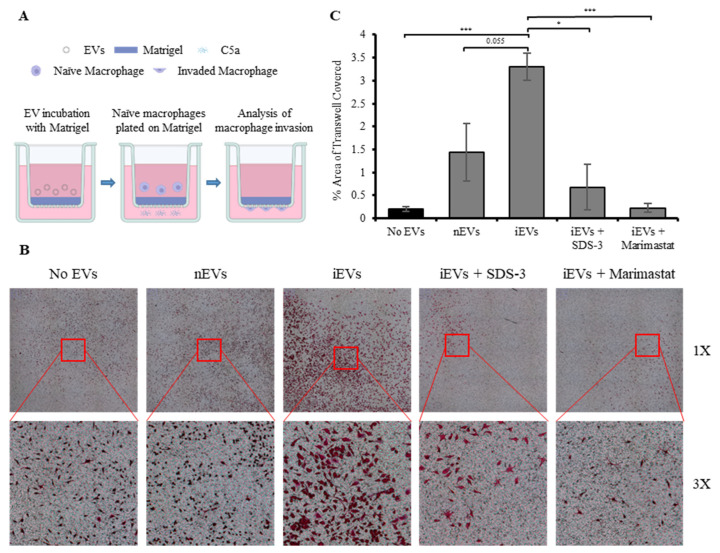
EV-associated MMP-9 derived from *Salmonella* Typhimurium-infected macrophages increases macrophage invasion through simulated basement matrix. (**A**) Schematic illustration in vitro invasion assay. (**B**) Representative images of crystal violet-stained macrophages on the bottom half of Transwell inserts treated with PBS, iEVs, nEVs, and iEVs pre-treated with either 0.1 µM or 1 µM of SDS-3 or 1 µM Marimastat, respectively. (**C**) Image analysis quantifying the number of macrophages that have invaded through the Matrigel into the bottom half of Transwell inserts treated with PBS, nEVs, and iEVs pre-treated with either 0.1 µM or 1 µM of SDS-3 or 1 µM Marimastat, respectively. * *p* < 0.05, *** *p* < 0.001.

To investigate potential functions of macrophage-derived EV-associated MMP-9, an in vitro invasion assay was performed (Figure 6A). Transwell inserts with a pore size of 8 µm were coated with Matrigel, a solubilized basement membrane matrix, as described in the Materials and Methods section. After gelling, the Matrigel was incubated with 10 µg of nEVs or iEVs obtained from macrophages infected with *S.* Typhimurium at an MOI of 50. The iEVs were pre-incubated for 2 h with either PBS, or with 0.1 µM of SDS-3 [82], a selective inhibitor of MMP-9 and MMP-2, or with 1 µM of Marimastat, a small molecule broad-spectrum MMP inhibitor. The EVs, normalized by total protein amount, were incubated with the Matrigel-coated Transwell inserts for 4 h. Afterward, the media containing the EVs was removed, and 10^5^ naïve macrophages were introduced to the top half of the Transwell insert. After 36 h the Transwell inserts were removed, washed, and fixed in 4% PFA, followed by staining with crystal violet solution. The invading macrophages were imaged using a Widefield microscope, and the number of stained macrophages was quantified using ImageJ software v1.54d. Representative images of the Transwell inserts from the different treatments are shown (Figure 6B). Image analysis revealed that treatment of the Matrigel with iEVs increased the number of naïve macrophages invading the bottom half of the Transwell insert more than 3-fold compared to Matrigel pre-incubated with no EVs, and more than 2-fold compared to Matrigel pre-incubated with nEVs (Figure 6C). Furthermore, pre-treating the iEVs with either SDS-3 or Marimastat significantly reduced the number of invading macrophages, indicating that macrophage-derived EV-associated MMP-9 substantially enhances the ability of naïve macrophages to invade through the basement matrix.

### 3.8. EV-Associated MMP Activity Drives Increased EV Uptake in the Spleen and Liver

**Figure 7:**
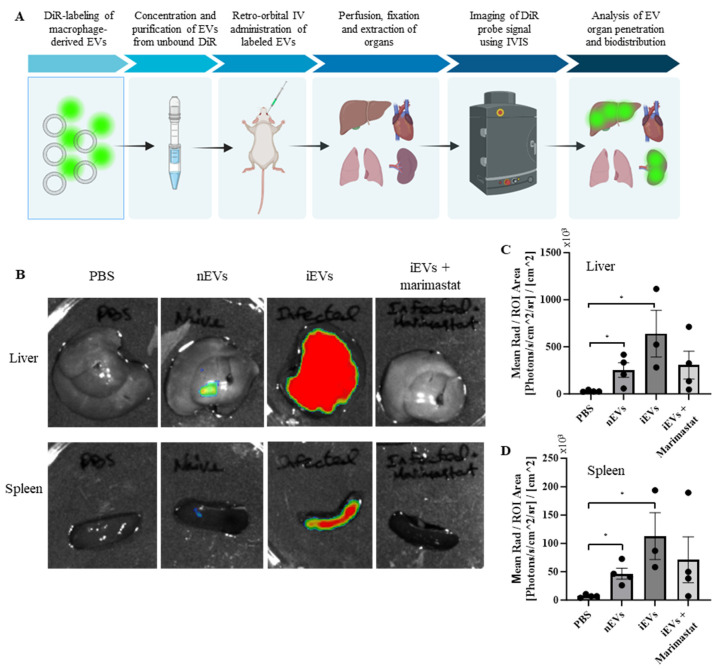
EV-associated MMP activity drives EV uptake into the spleen and liver. (**A**) Schematic illustration of the EV in vivo biodistribution experiment. (**B**) Representative images of the spleens and livers of mice treated with PBS, DiR-labeled EVs from naïve macrophages, and DiR-labeled EVs from *S.* Typhimurium-infected macrophages, pre-treated with either PBS or 1 µM Marimastat, respectively. In the images, low fluorescent intensity values are represented in blue, intermediate values in yellow, and high fluorescent intensity signals in red. (**C**,**D**) Image analysis quantifying the fluorescence intensity of the livers and spleens, respectively. Mean radiance [Photons/s/cm^2^/sr] was normalized by ROI area [cm^2^]. Analysis was performed using the Aura Imaging Software v4.0.8. * *p* < 0.05.

To evaluate the ability of the macrophage-derived EVs to penetrate into organs, an in vivo EV biodistribution assay was performed (Figure 7A). As described above, EVs were isolated from either naïve macrophages or from *S.* Typhimurium-infected macrophages. The *S.* Typhimurium-infected macrophages were either pre-treated with PBS or with 1 µM Marimastat to inhibit EV-associated protease activity. Subsequently, the EVs were labeled and 4 × 10^10^ EVs from each group were injected retro-orbitally into male immunodeficient Hsd:Athymic Nude-Foxn1nu mice. After 24 h the mice were perfused and fixed in 4% PFA, and their brains, lungs, hearts, livers, spleens, kidneys, and intestines were excised. Fluorescent intensity was measured using IVIS imaging and analyzed using the Aura Imaging Software v4.0.8, as previously demonstrated [83]. To control for background fluorescence, mice were injected with PBS. No fluorescent signal was detected in the brains, lungs, heart, kidneys, and intestines of the nEV-treated mice over that of the PBS-treated mice. However, fluorescent signals were observed in the spleens and livers of both nEV- and iEV-treated mice (Figure 7B–D). The mice injected with the iEVs showed a marked increase in fluorescent signal intensity compared to those injected with the nEVs. Furthermore, this effect was eliminated in the mice injected with the iEVs pre-treated with Marimastat, resulting in fluorescence similar to that of the nEV-injected mice.

## 4. Discussion

Perturbations in EV-mediated communication between immune cells have been associated with both local and systemic inflammation [84,85], cancer [86], and neurodegeneration [52,53]. Extensive research has explored the involvement of EVs in the pathogenesis of various diseases and tissue regeneration, particularly focusing on their nucleic acid and proteomic contents. However, the secretion and biological activities of EV-associated matrix-remodeling enzymes and their regulators have only recently come to the fore. Existing literature extensively documents the pivotal role of proteolysis in facilitating the migration of immune cells through the ECM and the basement membrane toward infection sites. MMPs, whether membrane-bound or secreted, have been identified as key contributors to the proteolytic invasion process [87,88,89]. Recent studies have increasingly recognized the presence of EVs carrying proteases, including MMPs and ADAMs, primarily in the context of physiological processes, inflammation, cancer progression, and metastasis [40,41]. However, there remains a notable scarcity of literature addressing the role of EV-mediated matrix remodeling in infectious diseases.

To address this knowledge gap, we established an in vitro model of host–pathogen interaction by infecting J774a.1 murine macrophages with *S.* Typhimurium, a Gram-negative bacterium known for its effective infection of immune phagocytic cells.

A comprehensive MS-based proteomics analysis of EVs secreted by naïve and infected macrophages revealed 1192 differentially expressed proteins between nEVs and iEVs, indicating significant alterations in the protein cargo of macrophage-derived EVs following infection with *S*. Typhimurium. Intriguingly, pathway enrichment analysis identified the upregulation of glycosaminoglycan degradation and the collagen-associated pathway as the two most highly enriched pathways. These findings strongly suggest that iEVs play a crucial role in ECM remodeling. Of particular interest, a significant and specific upregulation of MMP-9 levels and its activity in iEVs was detected. Notably, the levels of its endogenous inhibitor, TIMP-2, were significantly downregulated.

Remarkably, the upregulation of MMP-9 expression and activity in macrophages upon exposure to both live and heat-killed *S*. Typhimurium indicates the involvement of an immune response to a bacterial component. Our results confirm early published reports in different experimental models [67,68,69], pointing to TLR-4-mediated signaling through an innate immune receptor for bacterial LPS as a regulator of this effect. Thus, the regulation of MMP-9 levels in iEVs occurs through mechanisms that regulate cellular expression and secretion of MMP-9 via LPS and TLR-4 signaling [68,69,72]. At the very least, partial localization of MMP-9 on the EV-membrane was confirmed by the detection of known complexes of MMP-9 with the LRP-1 scavenger receptor, recognized for its binding to MMP-9 for reuptake into the cell [73,74], and fibronectin, known for its ability to bind MMP-9 and present it on the cellular surface [75,76].

Remarkably, it was demonstrated that membrane-bound MMP-9 is more resistant to inhibition by TIMPs than its soluble form. Consequently, the localization of MMP-9 on the membrane surface of iEVs enhances its degradation activity, thus promoting ECM remodeling [90]. As expected, high proteolytic activity of iEVs was observed in an in vitro macrophage invasion assay using Matrigel-coated Transwell inserts. Interestingly, the invading capacity of macrophages was abolished when iEVs were pre-treated with a selective antibody inhibitor, SDS-3, or Marimastat, demonstrating the strong involvement of iEVs enriched with MMP-9 in ECM degradation. An in vivo EV biodistribution assay further confirmed the enhanced proteolytic ability of iEVs. In contrast to nEVs, they were preferentially taken up and predominantly distributed in the liver and spleen. Furthermore, pre-treatment of the iEVs with Marimastat markedly reduced the uptake of iEVs, indicating that active MMPs associated with macrophage-derived EVs play a crucial role in promoting EV accumulation in tissues from systemic circulation. This enhanced tissue accumulation of iEVs could potentially facilitate more efficient delivery of EV cargo, whether it comprises proteolytic enzymes or signaling molecules, or augments the capacity of macrophages to extravagate from the local vasculature and invade through the basement membrane and ECM to reach the site of infection. However, invasion and extravasation still need to be correlated with direct proteolysis to shed more light on the role of proteolytic enzymes associated with EVs.

This study strongly suggests that macrophages infected by *S.* Typhimurium secrete proteolytic EVs to increase invasion and ECM remodeling in bacterial infections. Further investigations are warranted to elucidate the mechanistic understanding of the involvement of MMP-9 in the increased invasiveness of EVs through the degradation of specific substrates. Understanding the interplay between proteolytic enzymes and EV cargo will provide valuable insights into the immunology and immunopathology of host–pathogen interactions. Finally, this study holds the potential to guide the development of EV-based diagnostics and therapeutics.

## Data Availability

Raw data and access to our data storage is available for all experiments per request.

## Supplementary Materials

The following supporting information can be downloaded at: https://www.mdpi.com/article/10.3390/biomedicines12020434/s1, Figure S1: Characterization of an in vitro model of macrophage-derived EVs; Figure S2: Validation of MMP-9 EV association via precipitation out of 20% sucrose cushion; Figure S3: *S*. Typhimurium-infected macrophage-derived EVs do not affect bacterial growth or infectivity; Table S1: Protein-protein interactions of EV-associated MMP-9.
