# Supplementary material for: Proteolytic Vesicles Derived from Salmonella enterica Serovar Typhimurium-Infected Macrophages: Enhancing MMP-9-Mediated Invasion and EV Accumulation
Source: Biomedicines. 2024 Feb 15;12(2):434. doi: 10.3390/biomedicines12020434 (PMC10886541; doi:10.3390/biomedicines12020434)
Supplement: Supplementary file 1 [file biomedicines-12-00434-s001.zip › biomedicines-2793550-supplementary.pdf]

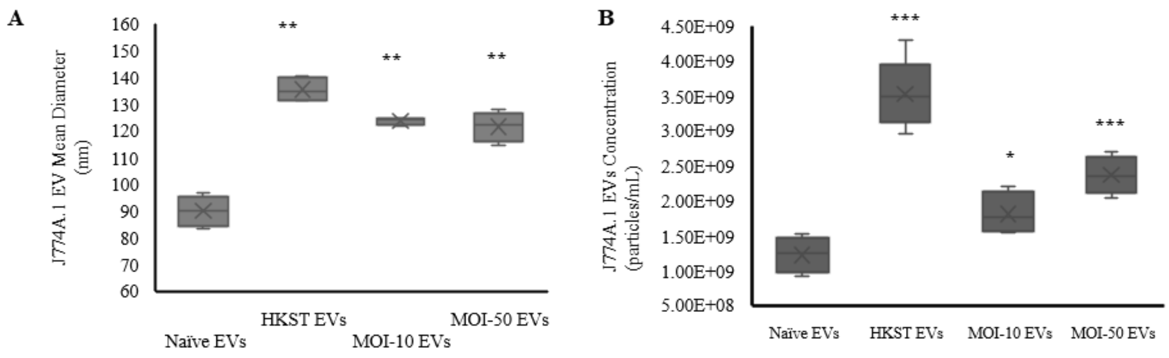

Supplemental Figure S1. Characterization of an *in vitro* model of macrophage-derived EVs. (A) Quantitative analysis of the mean diameters of macrophage-derived from naïve cells, cells exposed to heat-killed S. Typhimurium (HKST), or live *S*. Typhimurium at a MOI of 10 or 50, measured by nanoparticle tracking analysis (NTA). Each experiment was done in triplicates. (B) Quantitative analysis of macrophage-derived particle concentrations measured by NTA, showing the mean and error. EVs were collected from the same cells amount for each condition, and each experiment was conducted in triplicate.

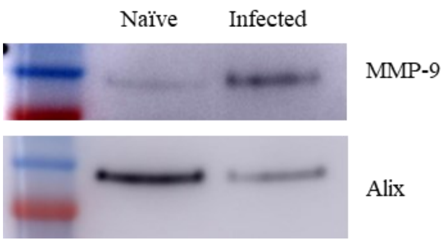

Supplemental Figure S2. Validation of MMP-9 EV association via precipitation out of 20% sucrose cushion. Representative Western blots of MMP-9 and Alix from EVs derived from naïve and S. Typhimurium-infected macrophages precipitated out of a 20% sucrose cushion.

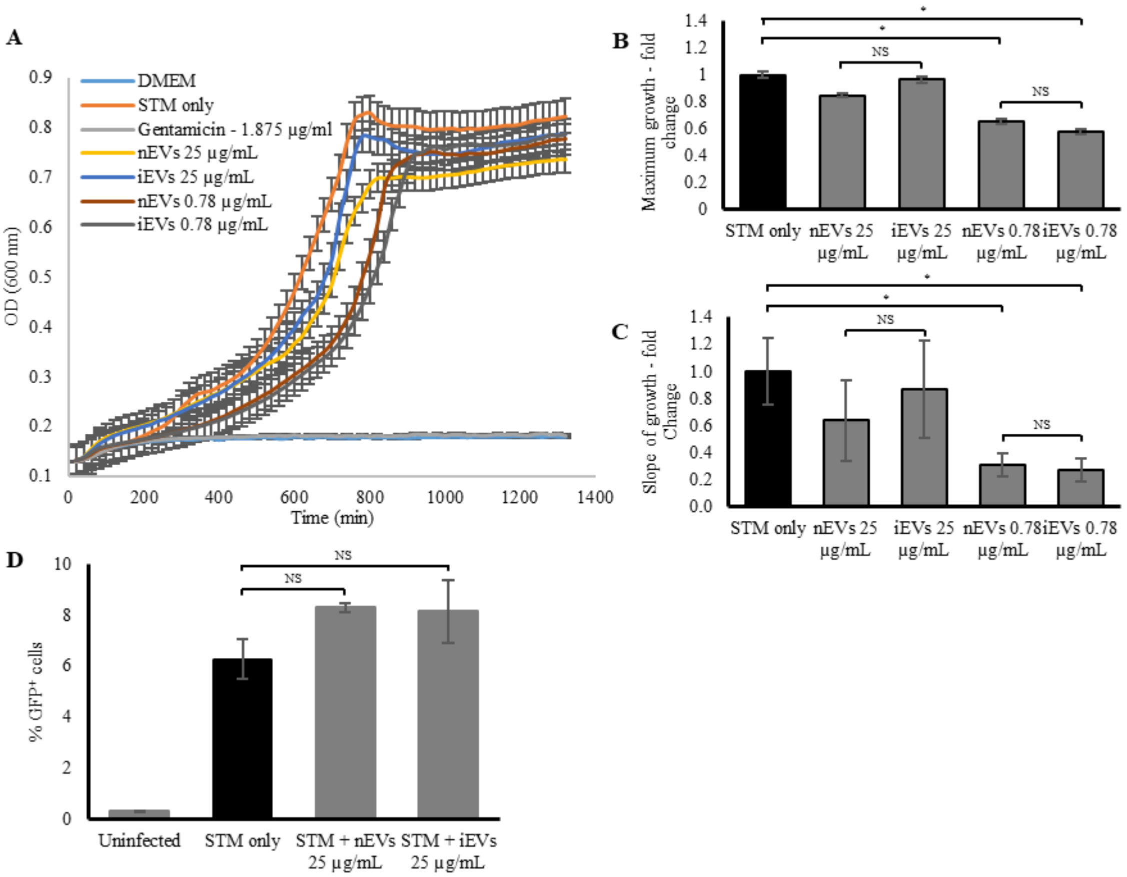

Supplemental Figure S3. S. Typhimurium-infected macrophage-derived EVs do not affect bacterial growth or infectivity. (A) Comparison of the growth curves of S. Typhimurium incubated with the highest dose (25 µg/mL) and low dose (0.78 µg/mL) of nEVs and iEVs. (B) Fold change of the maximum growth and (C) the maximum slope of growth of S. Typhimurium incubated with 25 and 0.78 µg/mL nEVs and iEVs, normalized to STM only. (D) Percentage of S. Typhimurium-infected Caco-2 epithelial cells pretreated with 25 µg/mL nEVs or iEVs. Each experiment was done in triplicates.

Protein names	Gene names	Log2 FC MMP9/IgG	Matrisome Category
Coiled-coil domain-containing protein 40	Ccdc40	13.42	
Matrix metalloproteinase-9	Mmp9	12.87	ECM Regulators
40S ribosomal protein S13	Rps13	8.23	
EMILIN-2	Emilin2	8.05	ECM Glycoproteins
Ig mu chain C region	Ighm	8.03	
Macrophage metalloelastase	Mmp12	7.83	ECM Regulators
Protein transport protein Sec31B	Sec31b	7.66	
Plasminogen;Plasmin heavy chain A;Activation peptide;Angiostatin;Plasmin heavy chain A, short form;Plasmin light chain B	Plg	7.43	ECM Regulators
Fibronectin;Anastellin	Fn1	7.16	ECM Glycoproteins
40S ribosomal protein S25	Rps25	6.70	
Programmed cell death 6-interacting protein	Pdcd6ip	6.49	
60S ribosomal protein L17	Rpl17	5.93	

40S ribosomal protein S19	Rps19	5.91	
Annexin A2;Annexin	Anxa2	5.88	ECM-affiliated Proteins
60S ribosomal protein L4	Rpl4	5.78	
RNA-binding protein with serine-rich domain 1	Rnps1	5.67	
40S ribosomal protein S20	Rps20	5.51	
60S ribosomal protein L24	Rpl24	5.50	
40S ribosomal protein S11	Rps11	5.49	
60S ribosomal protein L27a	Rpl27a	5.22	
T-complex protein 1 subunit beta	Cct2	5.01	
Prolow-density lipoprotein receptor-related protein 1;Low-density lipoprotein receptor-related protein 1 85 kDa subunit;Low-density lipoprotein receptor-related protein 1 515 kDa subunit;Low-density lipoprotein receptor-related protein 1 intracellular domain	Lrp1	5.01	
60S ribosomal protein L9	Rpl9	4.79	
Complement C4-B;Complement C4 beta chain;Complement C4 alpha chain;C4a anaphylatoxin;Complement C4 gamma chain	C4b	4.73	
60S ribosomal protein L21	Rpl21	4.68	
40S ribosomal protein SA	Rpsa	4.61	
60S ribosomal protein L28	Rpl28	4.43	
Otopetrin-2	Otop2	4.42	
Desmoplakin	Dsp	4.41	
40S ribosomal protein S4, X isoform;40S ribosomal protein S4	Rps4x	4.40	
Junction plakoglobin	Jup	4.26	
Serine/arginine-rich splicing factor 7	Srsf7	4.21	
Uridine phosphorylase 2;Uridine phosphorylase	Upp2	4.13	
40S ribosomal protein S23	Rps23	4.12	
Annexin A1;Annexin	Anxa1	4.11	ECM-affiliated Proteins
Clathrin heavy chain 1;Clathrin heavy chain	Cltc	4.06	
40S ribosomal protein S9	Rps9	4.02	
Lysozyme;Lysozyme C-1	Lyz1	4.00	

Supplemental Table S1. Protein-protein interactions of EV-associated MMP-9. EV lysates were used for immunoprecipitation using antibodies against MMP-9 or IgG. The immune complexes were subjected to mass spectrometry analysis. Interacting proteins showing log2 ratio >4 in MMP9 compared to IgG are presented.
